# Prevalence of renal insufficiency and factors associated among selected cancer patients on chemotherapy at Ocean Road Cancer Institute in Tanzania: a cross-sectional study

**DOI:** 10.1186/s12885-024-12419-y

**Published:** 2024-06-25

**Authors:** Hamidu N. Rajabu, Sven Gudmund Hinderaker, Penina Mnandi, Ritah F. Mutagonda

**Affiliations:** 1 Department of Pharmacy, Kilimanjaro Christian Medical Centre, Moshi, Tanzania; 2 Department of Clinical Pharmacy and Pharmacology, Muhimbili University of Health and Allied Sciences, Dar-es- salaam, Tanzania; 3 Department of Global Public Health and Primary care, University of Bergen, Bergen, Norway; 4 Department of Pharmacy, Ocean Road Cancer Institute, Dar-es- salaam, Tanzania

**Keywords:** Renal insufficiency, Creatinine clearance, Cancer, Chemotherapy, Ocean Road Cancer Institute

## Abstract

**Background:**

Cancer is among the leading cause of death worldwide. Chemotherapy is commonly used in cancer management and among the challenges in managing cancer patients is renal insufficiency (RI), which can be due to cancer or anticancer treatment and can be potentiated by different factors. Data regarding the prevalence of RI and associated factors in Tanzania is scanty. This study aims to assess the prevalence of RI and associated factors among selected cancer patients on chemotherapy.

**Methods:**

This analytical cross-sectional study was conducted at Ocean Road Cancer Institute (ORCI) in Dar es Salaam, Tanzania, from March to May 2023. The study included cancer patients on chemotherapy. Data was collected using semi-structured questionnaires whereby socio-demographics, clinical and laboratory data were recorded. Data was analyzed by using STATA version 15. Categorical data was presented as frequencies and percentages, and continuous data was summarized using means. A modified Poisson regression model was used to assess factors associated with RI. The *p*-values ≤ 0.05 was considered statistically significant.

**Results:**

Out of 354 patients, the majority (76.6%) were female. The enrolled patients’ mean age was 53 ± 13.19 years. The proportion of cancer patients with RI was 62.2% with most (60%) having stage 2 and stage 3 (37.7%). Age, hypertension (HTN), human immunodeficiency virus (HIV), diabetes mellitus (DM) and non-steroidal anti-inflammatory drugs (NSAIDs) use were significantly associated with increased risk of RI (*p* ≤ 0.05).

**Conclusion:**

This study showed that RI is common among cancer patients on chemotherapy. Age, HTN, DM, HIV and NSAIDS use were associated with RI. Close monitoring of kidney function is necessary for cancer patients with other factors associated with RI. Use of creatinine clearance (CrCl) rather than serum creatinine in estimating kidney function is important.

## Background

Cancer is among the leading causes of death worldwide in developing and developed countries, whereby 10 million deaths occurred worldwide in 2020, and 26,945 deaths in Tanzania [1]. The most prevalent types of cancer worldwide are breast, lung, colon, and prostate, while in Tanzania, the leading cancers are cervical, breast, prostate, and colorectal [1]. There are different cancer treatment modalities; however, the most used is chemotherapy, which is not specific to cancerous cells and can attack even healthy cells. Renal insufficiency (RI) is common among cancer patients, which can limit proper treatment of the underlying malignant. Different studies have reported a high prevalence of RI among cancer patients on chemotherapy [2–5], a study conducted in Belgium by Janus et al. (2010) reported a prevalence of 64% of RI [6]. In cancer patients, RI can be caused directly by disease itself, immunoreaction (mechanisms of cell-mediated immune injury), or anticancer treatments [5, 6].

RI in cancer patients can also be potentiated by other factors, such as extracellular volume depletion, urinary tract obstruction, use of nephrotoxic drugs, and other comorbidities, such as HIV, diabetes, and hypertension [2]. RI interferes with the elimination of chemotherapy drugs, which could result in the accumulation of these drugs in the body; as a result, causing adverse drug events (ADEs) in patients [7, 8]. Clinical care of these patients with RI involves dosage adjustment due to changes in drug pharmacokinetics and close monitoring of kidney function before and after initiation of chemotherapy [9, 10]. To our knowledge, there is not enough information on the prevalence of RI among cancer patients on chemotherapy and associated factors in Tanzania. Therefore, this study aims to assess the prevalence of RI and associated factors among cancer patients on chemotherapy at Ocean Road Cancer Institute (ORCI).

## Methods

### Study design, site, and population

This hospital-based analytical cross-sectional study was conducted on cancer patients at ORCI in Dar es Salaam, Tanzania, from March to May 2023. ORCI is the only comprehensive specialized facility for cancer care in Tanzania. The Institute serves over 50,000 patients including about 28,000 cancer patients, 10,000 cancer screening patients, and 12,000 non-cancer patients. ORCI offers laboratory services, diagnostic imaging, chemotherapy, radiotherapy, palliative care, cancer screening, and HIV care.

Patients included in this study were those with prostate, breast, colorectal, and cervical cancer, 18 years old and above, who were on chemotherapy and were willing to give informed consent for participation. Patients were excluded if they were on renal replacement therapy, critically ill, or if there was missing information in the patient files such as current serum creatinine levels because without this data it is impossible to estimate the CrCl however in this study no patient was excluded because of missing information.

A total of 354 participants were enrolled in this study. The sample size was calculated using a standard formula, a prevalence from the previous study of 64% of RI among cancer patients was used [6], a confidence level of 95%, and a margin of error of 5% were applied. The sampling strategy in this study was non-probability consecutive, whereby consent was requested from all patients who met inclusion criteria and recruited until the sample size was reached.

### Data source and collection

Prior to enrolment, informed consent was acquired for those who accepted to participate in our study. A semi-structured questionnaire included socio-demographics, co-morbidities, and medication history were asked from all patients and clinical and laboratory data such as complete blood count (Hemoglobin, white blood cells and platelets), renal function test, cancer type, the current and previous chemotherapy drugs including the type of chemotherapy, total dose, route, number of cycles, length of a cycle, and current cycle were obtained from patients’ files.

### Variable definition

The primary outcome was RI, measured as the presence or absence of RI based on creatinine clearance (CrCl), which was calculated from the serum creatinine (SCr) level (The recent pre-chemotherapy investigation during the chemotherapy session before administration for those who are on chemotherapy) using a Cockcroft-Gault formula.

$$\begin{gathered} C{r_{Cl}}\left( {ml/min} \right) = \left\{ {\left( {\left( {140  - age} \right){\text{ x }}weight} \right)/\left( {72x{S_{Cr}}} \right)} \right\}{\text{x}} \hfill \\\,\,\,\,\,\,\,\,\,\,\,\,\,\,\,\,\,\,\,\,\,\,\,\,\,\,\,\,\,\,\,\,\,\,\,{\text{ }}0.85{\text{ }}\left( {if{\text{ }}female} \right) \hfill \\ \end{gathered}$$

Participants with CrCl less than 90 ml/min were considered to have RI, and they were classified into different stages according to Kidney Disease Improving Global Outcomes (KDIGO) [11].

### Statistical analysis

Data was analysed using STATA software version 15, whereby categorical data were presented as frequencies and percentages, and continuous data were summarized using median or mean values depending on data distribution. Modified Poisson regression was used to assess factors associated with RI, whereby univariable and multivariable analyses were done. After univariable analysis, factors with a *p*-value of less than 0.2 were analyzed in multivariable analysis. Variables with *p*-values < 0.05 were considered statistically significant predictors of RI.

## Results

### Patients baseline characteristics

Table 1 below describes the baseline characteristics of enrolled participants, whereby 354 patients were included, and 271 (76.6%) were female. The mean age was 53 ± 13.19 years, and more than half, 193 (54.5%), were middle-aged adults (40–59) years. Most patients, 333 (94.1%) never smoked and 284 (80.2%) never used an alcohol. A total of 116 (32.8%) patients had co-morbidities such as HTN (24.01%), HIV (8.2%), DM (4.8%) and CVD (1.1%). More than half (59.9%) of the patients had no anaemia, and 26 (7.3%) had high serum creatinine.

**Fig. 1:**
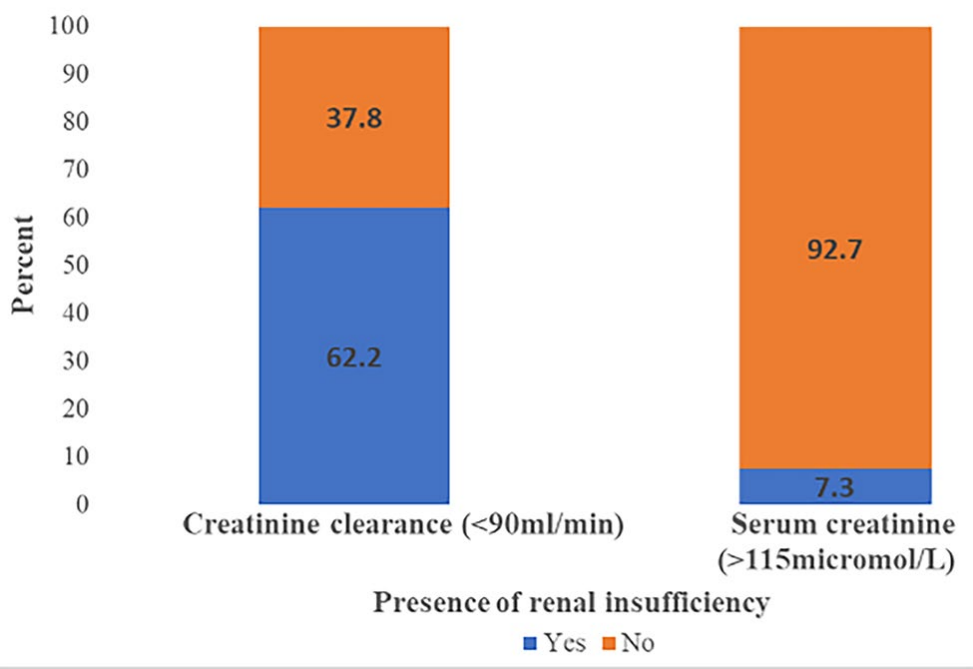
Proportion of patients with renal insufficiency based on creatinine clearance and serum creatinine

The most commonly occurring cancers were breast 142 (40.1%) and cervical cancer 111 (31.4%). Most of them, 143(40.4%), had stage II cancer disease, and only 81 (22.9%) of the patients were also on radiotherapy. Among 354 patients, 220 (62.2%) had RI based on CrCl and only 7.3% had RI based on SCr (Fig. 1).

**Fig. 2:**
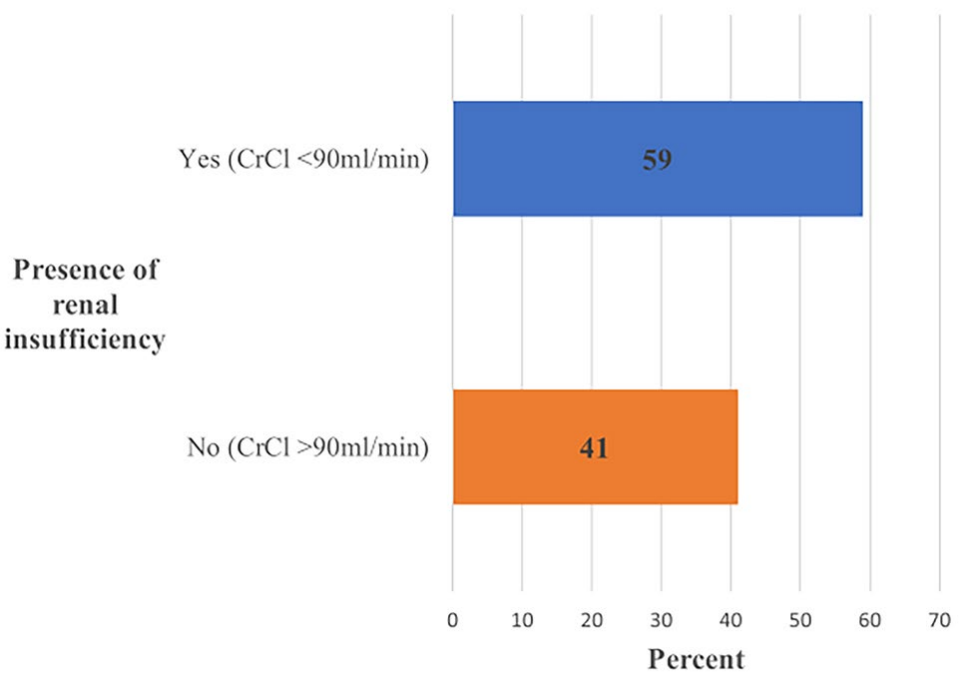
Proportion of patients with renal insufficiency who presented with normal serum creatinine (< 115micromol/L)

A high proportion of patients with renal insufficiency is seen when estimated based on creatinine clearance (< 90 ml/min) comparing to serum creatinine. Of 328 patients with normal serum creatinine, 59% were presented with RI when estimated using creatinine clearance (Fig. 2).

**Table 1:** Baseline characteristics of patients (*N* = 354)

Variable	*n*	%
**Gender**		
Female	271	76.6
Male	83	23.4
**Age group (years)**		
Young adults (18–35)	53	14.9
Middle aged adults (40–59)	193	54.5
Old adults (60 and above)	108	30.5
The mean age in years ± SD 53 ± 13.19 **Smoking status**		
Ex smoker	21	5.9
Never	333	94.1
**Alcohol use**		
Drinking	2	0.6
Ex drinker	68	19.2
Never	284	80.2
**Insurance status**		
Covered	179	50.6
Not covered	175	49.5
**Serum creatinine**		
Normal (45-115micromol/L)	328	92.7
High (> 115micromol/L)	26	7.3
**Other comorbidities**		
Yes	116	32.8
No	238	67.2
**Type of comorbidity**		
Hypertension	85	24.0
Diabetes mellitus	17	4.8
Cardiovascular disease	4	1.1
HIV	29	8.2
**Cancer type**		
Breast	142	40.1
Cervical	111	31.4
Colorectal	63	17.8
Prostate	38	10.7
**Cancer stage**		
I	7	1.9
II	143	40.4
III	83	23.5
IV	121	34.2
**Radiotherapy**		
Yes	81	22.9
No	273	77.1
**Type of chemotherapy**		
Alkylating agents	84	23.7
Alkylating agents + antimicrotubular	37	10.5
Alkylating agents + antibiotics	51	14.4
Alkylating agents + antimetabolites	65	18.4
Antimicrotubular	106	29.9
Others	11	3.1
**Presence of renal insufficiency**		
Yes	220	62.2
No	134	37.8
**Stages of renal insufficiency**		
Stage 2 mildly decreased (60–89 mL/min)	132	60.0
Stage 3 moderately decreased (30–59 mL/min)	83	37.7
Stage 4 severely decreased (15–29 mL/min)	5	2.3

Of 354 patients, 121 (34.2%) had a previous history of chemotherapy use,112 (31.6%) had a history of herbal medication use, and 288 (81.4%) had a history of NSAID use. Others reported use of proton pump inhibitors (PPI) (36.7%), angiotensin receptor blockers (ARB) (8.2%), antiretroviral therapy (ART) (7.1%) and diuretics (4.2%) (Table 2).

**Table 2:** Medication history of patients (*N* = 354)

Variable	*n*	%
**History of chemotherapy use**		
Yes	121	34.2
No	233	65.8
**History of herbal drugs**		
Yes	112	31.6
No	242	68.4
**History of NSAIDs use**		
Yes	288	81.4
No	66	18.6
**History of PPIs use**		
Yes	130	36.7
No	224	63.3
**History of ART use**		
Yes	25	7.1
No	329	92.9
**History of ARB use**		
Yes	29	8.2
No	325	91.8
**History of diuretics use**		
Yes	15	4.2
No	339	95.8

Table 3 below represents a univariable, and multivariable analysis of factors associated with RI whereby, after adjusting for confounders, the factors associated with RI are elderly age, HTN, HIV, DM and NSAIDs use.

**Table 3:** Factors associated with renal insufficiency (*N* = 354)

Variable	cPR (95% CI)	*p*-value	aPR (95% CI)	*p*-value		
**Age**						
60 and above	1.42(1.21–1.64)	< 0.001	1.41(1.20–1.64)	< 0.001		
< 60	Ref		Ref			
**Gender**						
Male	1.01(0.84–1.23)	0.914				
Female	Ref					
**Obesity**						
Yes	0.82(0.65–1.02)	0.072	0.91(0.73–1.13)	0.378		
No	Ref		Ref			
**Smoking**						
Ex smoker	0.91(0.63–1.34)	0.646				
Never	Ref					
**Alcohol use**						
Ex drinker	0.93(0.75–1.15)	0.507				
Never	Ref					
**Hypertension**						
Yes	1.33 (1.13–1.55)	< 0.001	1.29(1.05–1.60)	0.014		
No	Ref		Ref			
**Diabetes mellitus**						
Yes	0.56(0.29–1.07)	0.077	0.48(0.28–0.84)	0.009		
No	Ref		Ref			
**Anemia**						
Yes	1.19(1.02–1.41)	0.026	1.12(0.96–1.31)	0.146		
No	Ref		Ref			
**Metastasis**						
Yes	0.92(0.77–1.09)	0.344				
No	Ref					
**History of chemotherapy**						
Yes	1.09 (0.93–1.29)	0.273				
No	Ref					
**Radiotherapy**						
Yes	1.26 (1.07–1.49)	0.005	1.06 (0.78–1.45)	0.709		
No	Ref		Ref			
**HIV**						
Yes	1.51(1.29–1.75)	< 0.001	1.51(1.20–2.05)	0.001		
No	Ref		Ref			
**NSAIDs**						
Yes	1.87(1.35–2.60)	< 0.001	1.65(1.22–2.24)	0.001		
No	Ref		Ref			
**Herbal drugs**						
Yes	1.16(0.99–1.37)	0.069	1.09(0.94–1.29)	0.242		
No	Ref		Ref			
**PPI use**						
Yes	0.95(0.79–1.12)	0.531				
No	Ref					
**ART use**						
Yes	1.46(1.23–1.73)	< 0.001	0.89(0.67–1.18)		0.418	
No	Ref		Ref			
**ARB use**						
Yes	1.56 (1.37–1.79)	< 0.001	1.19(0.93–1.53)		0.150	
No	Ref		Ref			
**Diuretics use**						
Yes	1.42(1.14–1.76)	0.002	1.04(0.77–1.41)		0.783	
No	Ref		Ref			
**Type of chemotherapy**						
Alkylating agents	1.04(0.85–1.29)	0.682	1.09(0.78–1.54)		0.600	
Alkylating + antibiotics	0.65(0.46–0.91)	0.011	0.86(0.62–1.19)		0.367	
Alkylating + antimetabolites	0.82(0.63–1.07)	0.147	0.92(0.72–1.19)		0.552	
Antimicrotubular agents	0.75(0.59–0.96)	0.021	0.83(0.65–1.07)		0.146	
Others	Ref		Ref			

Key: cPR: crude Prevalence Ratio, aPR: adjusted Prevalence Ratio

## Discussion

Occurrence of RI among cancer patients on chemotherapy is common as reported by previous studies [2–4]. However, in Tanzania there is limited information on prevalence of RI and associated factors among cancer patients. Therefore, this hospital-based analytical cross-sectional study was conducted to assess the prevalence of RI and associated factors among selected cancer patients on chemotherapy. This study found a high prevalence of RI among cancer patients on chemotherapy whereby 62.2% of the patients had RI after estimation of renal function by using CrCl. This finding is supported by different studies worldwide, which have reported high prevalence of RI among cancer patients on chemotherapy [10]. A study in Belgium by Janus et al. (2010) reported a prevalence of 64% of RI among cancer patients. Another study in Brazil by Pontes et al. (2014) reported a high prevalence (66%) of RI [4, 6], and also a study that was done in Zimbabwe by Manyau et al. (2021) in cervical cancer patients on cisplatin reported a high prevalence (43%) of RI among these patients [5]. The high prevalence of RI among cancer patients might be due to the disease (progression and metastasis) or anticancer treatments (chemotherapy and radiotherapy) [12–15]. More than half of our patients (59%) with normal SCr had unrecognized RI after estimation using CrCl. This is comparable to a study by Launay et al. (2010) on breast cancer, which reported more than 50% of the patients had unrecognized RI after estimate using GFR using the Cockcroft -Gault formula, but these patients had a normal SCr [16]. Many other studies have also reported a high prevalence of unidentified RI [9, 17, 18]. A patient with a CrCl of less than 90 ml/min is generally considered to have RI. However, in practical terms, if the CrCl falls below 60 ml/min, it is advisable to contemplate dose adjustments for chemotherapies primarily excreted through the kidneys. Additionally, it is recommended to avoid nephrotoxic chemotherapies in such cases [10].

In this study, females were more predominant which is comparable to other studies worldwide [6, 19, 20]. This is due to the fact that, most of our patients had breast and cervical cancer and in Tanzania the most prevalent cancers are cervical, breast and prostate [21], but it is contrary to a study in India which reported lungs, mouth and tongue cancer as the common ones, which could be due to difference in ethnicity and exposure to risk factors [22]. Among patients with comorbidities, HTN was the most common (24%). This is comparable to a study in England that found HTN to be common among cancer patients whereby 20% of the patients were having HTN [23]. The presence of comorbidities such as HTN, DM, and HIV is among the risk factors associated with RI, and cancer patients with these comorbidities need close monitoring of kidney function [24, 25].

Our study found different factors associated with RI; among the factors is age, whereby this study found that elderly patients are 1.4 times more likely to have RI compared to non-elderly patients, this is because ageing is characterized by a decline in renal function [26]. This is comparable to other studies that have been done in the USA and Belgium, which reported the same findings that age was associated with the occurrence of RI among cancer patients [6, 27]. Other factors that were found to be associated with RI were HTN, HIV, DM and NSAIDs use. Different articles have reported the influence of these factors on the development of RI [25, 28]. The prevalence of RI was 1.3 times more likely among cancer patients with HTN compared to non-hypertensive cancer patients. HTN can lead to nephron damage hence reducing its number, which can accelerate the progression of kidney disease [29]. In HIV patients, the prevalence of RI was 1.5 times more likely compared to non-HIV patients [30]. Also, our study found that cancer patients who used NSAIDs are 1.7 times more likely to have RI compared to those who didn’t use NSAIDs. Different articles have reported the influence of prolonged use of NSAIDs on kidney damage [31, 32]. These findings from our study are comparable to different studies which reported these factors to be associated with RI [25, 28]. Some studies also found other factors such as hypercalcemia, sepsis and dyslipidemia which are associated with RI, but in this study we were unable to document this information [28, 33]. In this study one of the limitation was the difficulty in determining whether the outcome (RI) followed risk factors in time or risk factors resulted from the outcome since it was a cross sectional study and some of the risk factors such as use of bisphosphonates or contrast agents were not assessed due to difficult in getting this information.

## Conclusion

This study showed that RI is common among cancer patients on chemotherapy. The study’s findings are crucial, revealing unrecognized RI in our setting. Age, HTN, Diabetes, HIV and NSAIDS use were associated with RI. Close monitoring of renal function is important in all cancer patients, but particularly in patients with those factors shown to be associated with RI. Use of CrCl rather than serum creatinine in estimating kidney function is important. It is essential to emphasize the necessity for dose adjustment in cancer patients with RI using chemotherapies that are potentially nephrotoxic or principally excreted renal.

## Data Availability

Data will be available upon request to a corresponding author.

## Abbreviations

ARB: Angiotensin receptor blocker
CKD: Chronic kidney disease
CrCl: Creatinine clearance
DM: Diabetes Mellitus
HTN: Hypertension
KDIGO: Kidney Disease Improving Global Outcomes
RI: Renal insufficiency
SCr: Serum creatinine
